# Task-Related Effects on the Temporal and Spatial Dynamics of Resting-State Functional Connectivity in the Default Network

**DOI:** 10.1371/journal.pone.0013311

**Published:** 2010-10-13

**Authors:** Omer Grigg, Cheryl L. Grady

**Affiliations:** 1 Rotman Research Institute at Baycrest, Toronto, Ontario, Canada; 2 Department of Psychiatry, University of Toronto, Toronto, Ontario, Canada; 3 Department of Psychology, University of Toronto, Toronto, Ontario, Canada; Indiana University, United States of America

## Abstract

Recent evidence points to two potentially fundamental aspects of the default network (DN), which have been relatively understudied. One is the temporal nature of the functional interactions among nodes of the network in the resting-state, usually assumed to be static. The second is possible influences of previous brain states on the spatial patterns (i.e., the brain regions involved) of functional connectivity (FC) in the DN at rest. The goal of the current study was to investigate modulations in both the spatial and temporal domains. We compared the resting-state FC of the DN in two runs that were separated by a 45 minute interval containing cognitive task execution. We used partial least squares (PLS), which allowed us to identify FC spatiotemporal patterns in the two runs and to determine differences between them. Our results revealed two primary modes of FC, assessed using a posterior cingulate seed – a robust correlation among DN regions that is stable both spatially and temporally, and a second pattern that is reduced in spatial extent and more variable temporally after cognitive tasks, showing switching between connectivity with certain DN regions and connectivity with other areas, including some task-related regions. Therefore, the DN seems to exhibit two *simultaneous* FC dynamics at rest. The first is spatially invariant and insensitive to previous brain states, suggesting that the DN maintains some temporally stable functional connections. The second dynamic is more variable and is seen more strongly when the resting-state follows a period of task execution, suggesting an after-effect of the cognitive activity engaged during task that carries over into resting-state periods.

## Introduction

The default network (DN) is an ensemble of brain regions that shows deactivation during a wide range of externally-cued tasks compared to a task-free resting-state, and exhibits coherent low-frequency endogenous resting-state fluctuations [1]–[14]. Consequently, most studies of the DN have relied on either task-related deactivations or resting-state analysis to investigate its composition and function. In addition, these studies have mostly focused on the spatial domain, i.e., the regions that are part of the DN, and attempted to characterize changes in involvement of various DN regions, and in the strength of their connections due to age, disease, and other factors. Evidence has emerged from these studies that the DN modulates its spatial composition, and its functional connectivity (FC) pattern, under various conditions, such as varying levels of consciousness [15]–[17], and due to various syndromes, such as dementia and autism [5], [18]–[21].

An equally important question, and one that has direct relevance for cognitive studies, is whether the DN alters its functional structure across time or in response to cognitive activity, i.e. whether two resting-state scans that are separated by time or task execution will show the same spatial composition. Despite studies showing that the DN's general architecture appears to be stable and consistent across individuals [9], [22], there is evidence from a few studies that preceding tasks can affect the functional connectivity of the DN and other resting-state networks. For example, Waites et al examined functional connectivity in several resting networks, before and after a language task, and found increased connectivity between the posterior cingulate (PCC), a node of the DN, and medial frontal regions after the task [23]. Changes of connectivity in a network involving a language-related area in the left inferior frontal gyrus also were noted. Similarly, Duff et al [24] showed increased spectral power and inter-regional correlations in a resting network involving motor cortex after participants performed a series of motor tasks. Changes in resting connectivity of visually-selective brain areas, such as the fusiform face area, also have been noted after exposure to their preferred stimulus type, relative to a non-preferred type [25]. These studies indicate the malleable nature of the spatial characteristics of resting state networks, but there is still not much known about how the resting DN per se is influenced by cognitive tasks.

The temporal nature of the DN also has received some recent attention. Barnes et al [26] measured the fractal scaling properties of the DN during rest, as a measure of the low frequency oscillations of the BOLD signal, and found a decrease after performance of working memory tasks that gradually returned to pre-task levels, possibly reflecting post-task “recovery” of the resting-state. In addition, this recovery was slower after more demanding tasks than after easier ones. Chang and Glover [27] showed, using wavelet analysis, that there is some temporal variability in the resting-state FC of DN regions with the PCC when assessed over 12-15 minutes of rest. Greater variability was seen in those regions that correlated negatively with the PCC (e.g., areas related to networks engaged during tasks), relative to the DN regions that correlated positively with the PCC. These two studies suggest that the DN may dynamically alter its functional connections across relatively short time scales of minutes, or even seconds, either in response to a preceding task or endogenously.

The abovementioned preliminary evidence points to two potentially major DN characteristics that are in need of further investigation: the temporal dynamics of the network during rest, and possible task-related influences on the spatial FC patterns during the resting-state. However, no study to date has investigated modulations in both the spatial and temporal domains; this was the goal of the current study. We compared the resting-state FC of the DN in two runs that were separated by a 45 minute interval containing cognitive task execution. We used partial least squares (PLS) for this purpose, which allowed us to identify FC spatiotemporal patterns [28] in the two resting runs and to determine differences between them. If the DN, as delineated during rest, is unaffected by any previous brain state, then both resting scans should have the same DN spatial pattern, as well as a constant FC measure through time. However, if the FC of the DN is influenced by cognitive activity, as we hypothesized, then the post-task connectivity should differ from that seen pre-task and could potentially involve changes in both DN regions and other areas involved in the cognitive tasks, for example task-positive network (TPN [7], [8]) regions, which are known to participate in a broad range of tasks. To distinguish changes in resting FC due to intervening cognitive activity from those that might be due to time in the scanner per se, we also compared FC in the first and second rest runs to that in the first and last task runs. This was done under the assumption that any change in FC seen in both rest and task runs might be due to time in the scanner, whereas changes seen only in the two resting runs would reflect an influence of the intervening cognitive activity.

## Methods

### Participants

Eighteen healthy right-handed young adults (age M = 24 years, SD = 3; 9 males) participated in this study after providing written informed consent. The Research Ethics Board of Baycrest Centre approved the study.

### Scanning Session

Each session included a high-resolution structural scan, followed by 10 functional runs, each lasting 5:40 minutes. The first and last runs were resting-state runs (Rest1 & Rest2), where subjects were instructed to lie still with their eyes closed, relax, and clear their minds, but to not actively suppress any thoughts that may spontaneously arise. Following scanning, subjects were asked if they fell asleep during the resting runs. Runs 2–9 were task runs, described below. Therefore, each session consisted of 2 resting-state runs, separated by 8 block-design runs of various tasks, a gap of about 45 minutes.

Each of the eight task-runs was composed of alternating 20 sec blocks of task and rest [29]. We used four task types: self-reference, other-reference, vowel identification, and motor. In all tasks, participants were shown a word and instructed to make a two-choice response. In the self-reference task subjects decided whether a personality-trait word represented them or not, in the other-reference task subjects judged whether the word represented a person they know well, and in the vowel identification task subjects identified whether the third letter from the end of the word was a vowel. The possible answers for these three tasks were “yes” or “no”. In the motor task the word was irrelevant, and participants pressed button 1 or 2, depending on a number shown on the screen.

### Image acquisition and Preprocessing

Scanning was carried out with a Siemens Trio 3T scanner. Anatomical scans were acquired with a 3D MP-RAGE sequence (TR = 2 sec, TE = 2.63 msec, FOV = 25.6 cm^2^, 256×256 matrix, 160 slices of 1 mm thickness). Functional runs were acquired with an EPI sequence (170 volumes, TR = 2 sec, TE = 30 msec, flip angle  =  70°, FOV = 20 cm^2^, 64×64 matrix, 30 slices of 5 mm thickness). Pulse and respiration were measured during scanning.

Preprocessing was performed with AFNI [30] and consisted of physiological correction for pulse and respiration [31], slice-timing correction for the resting runs, rigid-body motion correction, spatial normalization to the MNI template (TT_avg152T1, resampling our data to 2×2×2 mm), and smoothing (full-width half-maximum, 6 mm). The time series of the CSF, white matter, and major blood vessels were sampled from ROIs and regressed out from the data.

Lastly, we temporally resampled all voxels' time series by dividing the time series into 30 “blocks” of 5 consecutive volumes each, normalizing each block to the first volume of that block, and then averaging all volumes of the block. This averaging produced an effective low-pass filter of 0.1 Hz and reduced temporal noise. Since respiratory and cardiac fluctuations were shown to bias time course correlations [32]–[34], many FC studies apply a low-pass filter to their data. We did not apply such a filter because (a) our temporal resampling effectively filtered the data, (b) we applied physiological correction for pulse and respiration, and (c) PLS calculates correlations across participants, rather than within-subject time course correlations, as many FC studies do. We consider the block normalization performed in PLS as an alternative to global signal removal, a controversial preprocessing step that has been shown to bias correlation/anti-correlation observations [35]–[37].

### Data analysis

#### General approach

We analyzed our data with partial least squares [28], [38], [39], a multivariate approach that robustly identifies group-level spatiotemporal activity patterns correlated to neuronal activity (seed-PLS). As a multivariate approach, PLS computationally assumes that cognitive processes are a result of the activity of integrated neural networks, rather than activity of independent brain regions. The PLS approach to FC is somewhat different from time course correlation approaches or other methods that assess the within-subject relation between regions. Instead, PLS assesses across-subject correlations, which provides an indication of the stability of the relations between regions, and provides complementary information [39], [40].

PLS starts by creating a matrix of the correlations, across subjects, between seed activity and all other brain voxels for each “block”. This matrix is decomposed using singular value decomposition (SVD) to identify latent variables (LVs), which are orthogonal patterns of brain activity that characterize common or different patterns of group-level FC across “blocks”, thus assessing both spatial and temporal aspects of FC. For each LV, The SVD maximizes covariance and minimizes residuals between the seed activity and the spatiotemporal brain data. Each voxel has a weight, or salience, which is proportional to the covariance of its activity with the FC pattern on each LV. The significance for each LV as a whole is determined with a permutation test. The rows of the data matrix are re-ordered a number of times (i.e. the no. of permutations, in our case - 1000), and each time the SVD creates a new set of LVs, and the amount of covariance accounted for by these permuted LVs is compared to that of the original LV. The original value is assigned a probability based on the number of times the values from the permuted data exceed the original value. This is the permuted p value, representing the significance of the LV.

The reliability of each voxel's salience is determined with a bootstrap test. Subjects are randomly resampled with replacement a number of times (i.e. the no. of bootstraps, in our case - 1000), and their standard errors [SE, 41] are calculated. The ratio of salience value to the standard error for each voxel, or bootstrap ratio (BSR), is a measure of voxel reliability. Unlike univariate analyses, saliences are calculated in a single analytic step, thus no correction for multiple comparisons is required (for further details on PLS, see [28]). Voxel saliences are also used to assess how robustly each subject exhibits each LV's spatial pattern for each block, by summing them to produce a “brain score”. To provide an assessment of seed FC, brain scores are correlated with the seed activity in each “block”; this provides a measure of the correlated activity between the seed and the whole-brain pattern identified by the LV across time. The bootstrap is used to calculate confidence intervals around these correlations.

To investigate the whole-brain FC of the DN, we performed several seed-PLS analyses using a PCC seed (−2, −50, 28, a coordinate identified in a previous study [29]). Peak coordinates of regions that showed connectivity with the PCC were identified for all analyses using cluster reports with the following thresholds: cluster size = 80 voxels (0.64 ml), minimum distance between peaks = 1 cm, and a BSR equivalent to a two-tailed p value of 0.001 (i.e., BSR = 3.3). Within large clusters, multiple peaks were noted if they met the BSR threshold and were more than 1 cm from all other peaks. Anatomical labels were assigned using the Eickhoff Anatomy Toolbox [42] and an anatomy atlas [43].

### Pre-task vs. post-task resting-state DN FC

The first analysis included Rest1 and Rest2, and was a data-driven examination of the spatial and temporal characteristics of DN functional connectivity in the pre-task and post-task resting-states. We report here the temporal and spatial patterns of the two primary LVs, accounting for 42% of the covariance in the data (each of the remaining LVs accounted for <5% of the covariance). The spatial patterns in these two LVs were further assessed by calculating a series of conjunction maps, aimed at identifying the spatial commonalities and differences between them. To make these conjunction maps, the BSR maps of LV1 and LV2 were multiplied to create a new BSR image. Any voxel/cluster that had high BSR values in both LVs received an even higher BSR in the new map, thus it contained “hotspots” common to the two LVs. We then applied two different masks to this image to identify voxels with common or different patterns of FC, including only those voxels that met the BSR thresholds for each LV considered separately.

We next carried out a contrast-driven analysis, using specific contrasts entered into the analysis rather than using a data-driven approach. The purpose of this analysis was to identify regions with overall differences in the strength of FC between rest runs. For this analysis, we directly contrasted Rest1 and Rest2, across all time points, by entering a series of −1's for the Rest1 blocks and 1's for the Rest2 blocks.

### Comparing FC during rest and task

A second series of contrast-driven analyses was carried out to distinguish differences between rest runs that would be due to the influence of the intervening cognitive tasks from the effects of time in the scanner per se. These analyses included the first and last task runs, which we will refer to as Task1 and Task8, in addition to Rest1 and Rest2. These two task runs were separated by a temporal gap similar to the one separating the resting-state runs. The first contrast assessed a simple effect of time in the scanner (i.e. Rest1/Task1 vs. Task8/Rest2 = −1/−1/+1/+1) and the second assessed an interaction of time and type of run (Rest1/Rest2 = −1/+1 and Task1/Task8 = +1/−1, or together = −1 +1 −1 +1). That is, the second contrast was designed to identify those FC changes from Rest1 to Rest2 that differed from any change seen between the two task runs, and which could be attributed to some factor other than time in the scanner.

## Results

### Pre-task vs. post-task resting-state DN FC

**Figure 1:**
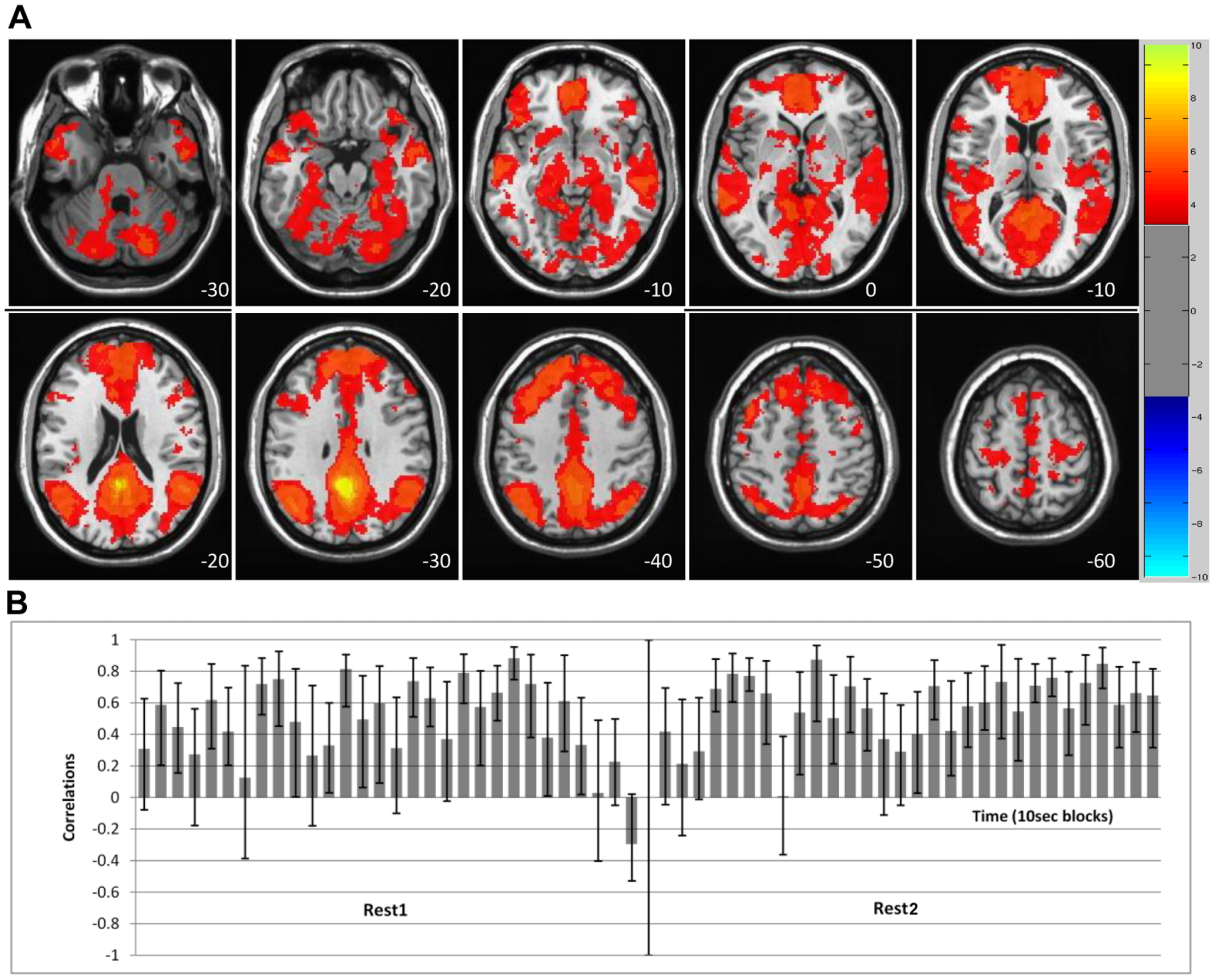
LV1 – the primary DN dynamic showing stable correlations. LV1 - The primary resting-state spatiotemporal pattern of PCC correlations, showing positive FC across most of the ‘blocks’ in both resting runs. A) The spatial composition, capturing the DN. The red regions (with positive BSRs) indicate areas with positive correlation with the PCC seed (no negative BSRs met the threshold, value range displayed is consistent with Figure 2). B) The temporal structure – correlations of brain scores with seed activity across time for each 10 sec ‘block’. Bars = 95% confidence intervals. The spatial and temporal correlational patterns are very similar across Rest1 and Rest2.

The data-driven analysis of Rest1 vs. Rest2 revealed two prominent spatiotemporal FC patterns for the PCC seed. The primary LV (36% of the covariance, p = 0.001) showed mostly positive correlations between the PCC and the rest of the DN across the entire run, for both Rest1 and Rest2 (Figure 1). DN areas with this pattern of FC included angular gyrus, middle temporal gyrus, medial temporal lobes, ventromedial frontal cortex, superior frontal gyrus, and cerebellum. Positive correlations were also found between the PCC and subcortical areas and the inferior frontal gyri (see Table 1 for the entire list). Only positive correlations with the PCC were seen on this LV.

**Table 1:** LV1 - Brain areas showing stable positive correlations with the PCC across time, for both Rest1 and Rest2.

Region	Hem	X(mm)	Y(mm)	Z(mm)	BSR
Angular gyrus	Left	−46	−62	30	20.98
Calcarine gyrus	midline	4	−90	6	11.60
Cerebellum	Left	−22	−78	−28	10.05
Cerebellum	Right	22	−42	−18	9.72
Cerebellum	Right	6	−50	−40	11.79
Cerebellum	Right	38	−56	−50	8.42
Fusiform gyrus – posterior region	Right	24	−82	−24	11.96
Inferior frontal gyrus p. orbitalis	Left	−42	26	−10	10.70
Inferior frontal gyrus p. triangularis	Left	−56	24	10	8.28
Inferior frontal gyrus p. triangularis	Right	58	30	8	9.36
Medial frontal gyrus	Left	−6	−28	66	8.43
Medial frontal gyrus	Left	−6	56	4	17.99
Medial frontal gyrus	midline	2	48	26	17.76
Middle frontal gyrus	Right	44	10	44	10.38
Middle temporal gyrus	Left	−66	−34	−2	13.72
Middle temporal gyrus	Left	−58	−14	−12	13.42
Middle temporal gyrus	Right	56	−28	−10	11.12
Middle temporal gyrus	Right	56	0	−22	13.03
**PCC**	midline	−2	−50	28	789.72
Precentral gyrus	Left	−18	−24	56	8.70
Precentral gyrus	Right	28	−26	54	7.69
Putamen/claustrum	Right	34	0	−12	10.10
SMA - BA6	Right	8	−10	66	7.50
Superior frontal gyrus	Left	−16	40	38	17.20
Superior frontal gyrus	Right	20	38	32	11.80
Superior temporal gyrus	Right	48	−52	20	19.05
Superior temporal gyrus	Right	42	26	−22	12.09
Thalamus	Left	−14	−18	−4	9.49

MNI coordinates. BSR>3.3 is equivalent to p<0.001. Hem = hemisphere; SMA = supplementary motor area; PCC = posterior cingulate cortex, the seed used in the FC analysis. See also Figure 1.

**Figure 2:**
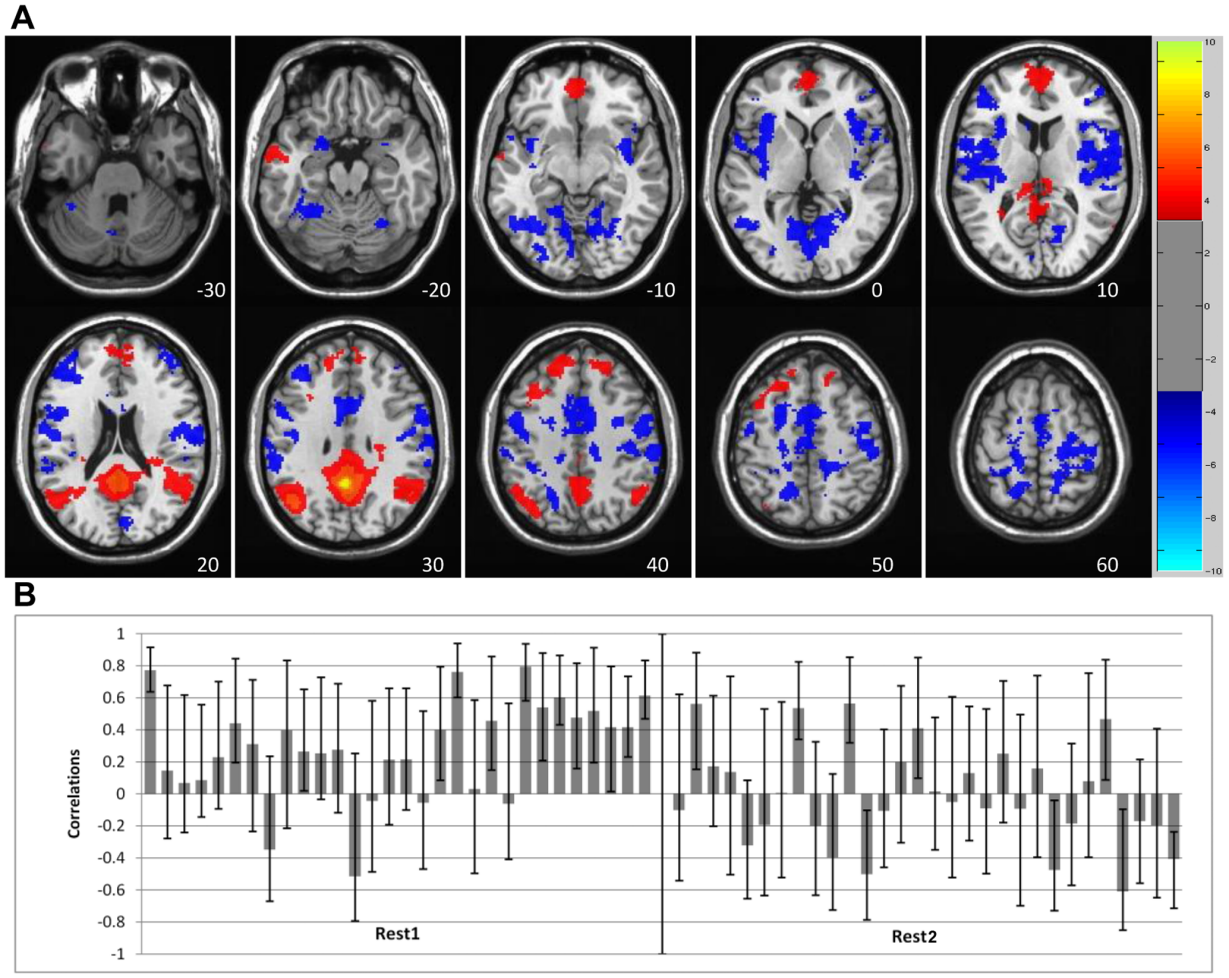
LV2 – the secondary DN dynamic showing variable correlations. LV2 - The secondary resting-state spatiotemporal pattern of PCC correlations, showing a transition from relative stability of DN connectivity to switching between two different patterns of FC. A) The spatial pattern of FC seen in this LV. Activity in red regions (positive BSRs) is associated with increased activity in the PCC during those blocks with positive correlations between brain scores and PCC (seen in B), whereas increased activity in blue areas (negative BSRs) is correlated with increased activity in the PCC for blocks where the correlations are negative. B) Correlations across time. Rest1 shows relatively stable positive correlations between the PCC and other DN regions, while Rest2 shows switching between the two patterns of connectivity. Bars = 95% confidence intervals for the correlations.

The secondary LV (6% of the covariance, p = 0.001) showed a pattern of mostly positive PCC connectivity during Rest1 with a subset of the regions seen in the first LV (red regions in Figure 2). However, during Rest2, the connectivity pattern was more variable across time. In some of the Rest2 blocks the PCC was positively correlated with the subset of DN regions that dominated the FC pattern during Rest1, and in other blocks was positively correlated with a different set of regions (blue regions in Figure 2, see Table 2 for the entire list). That is, during Rest2 the PCC switched its connectivity pattern back and forth between a group of DN regions and a different set of regions. Some of these other regions were similar to areas in the task-positive network (TPN, [7], [8]) that is often found to be negatively correlated with the DN. Indeed, a number of areas seen on LV2 were in close proximity (<1cm) to TPN regions reported by Fox et al [8], such as the anterior portion of the insula, precentral gyrus, and middle frontal gyrus. Thus, one way to think about LV2 is that it shows intermittent positive connectivity between the PCC and task-positive regions during Rest2.

**Table 2:** LV2 - Brain areas showing variable correlations with the PCC across time, during Rest2.

Region	Hem	X(mm)	Y(mm)	Z(mm)	BSR
Amygdala	Left	−18	2	−20	−5.69
Cerebellum	Left	−8	−72	−36	−5.57
Cerebellum	Right	32	−72	−50	−5.10
Cerebellum	Right	28	−46	−36	−3.84
Cuneus	midline	−4	−68	2	−7.19
Fusiform gyrus	Left	−30	−50	−18	−6.62
Inferior frontal gyrus p. triangularis	Left	−40	34	16	−6.66
Inferior frontal gyrus p. triangularis	Right	46	22	6	−6.09
Inferior occipital gyrus	Left	−32	−78	−8	−4.99
Inferior parietal lobule	Left	−62	−34	30	−5.23
Inferior parietal lobule	Right	24	−54	38	−4.03
Inferior parietal lobule	Right	62	−22	38	−5.30
Insula	Left	−30	18	6	−7.56
Insula	Left	−36	−4	10	−6.19
Insula	Right	38	−12	−2	−6.13
Middle cingulate cortex	Left	−6	6	40	−6.17
Middle cingulate cortex	Left	−8	−26	44	−5.22
Middle frontal gyrus	Right	32	0	38	−5.54
Middle frontal gyrus	Right	52	46	8	−5.10
Middle frontal gyrus	Right	42	46	20	−4.40
Middle frontal gyrus	Right	28	44	20	−5.19
Middle temporal gyrus	Right	56	−52	−6	−3.76
Postcentral gyrus	Left	−66	−18	30	−5.61
Postcentral gyrus	Right	60	−12	16	−7.00
Precentral gyrus	Left	−48	−2	46	−6.99
Precentral gyrus	Left	−56	6	4	−7.49
Precentral gyrus	Left	−20	−20	54	−6.61
Precentral gyrus	Right	58	2	28	−5.65
Precentral gyrus	Right	20	−26	58	−5.58
Precentral gyrus	Right	48	−4	50	−4.37
Precuneus	Left	−16	−58	48	−6.30
Precuneus	Right	14	−46	58	−5.77
Superior frontal gyrus	Left	−22	2	48	−6.99
Superior temporal gyrus	Left	−46	−18	6	−5.62
*Angular gyrus*	Left	−42	−64	28	12.07
*Angular gyrus*	Right	44	−48	26	9.32
*Medial frontal gyrus*	midline	2	54	−6	7.13
*Medial frontal gyrus*	midline	2	56	12	6.47
***PCC***	midline	−2	−50	28	398.19
*Superior frontal gyrus*	Left	−12	50	36	7.56

MNI coordinates. BSR>3.3 is equivalent to p<0.001. Hem = hemisphere; PCC = posterior cingulate cortex, the seed used in the FC analysis. Labels in *italics* are regions positively correlated with the seed. See also Figure 2.

**Figure 3:**
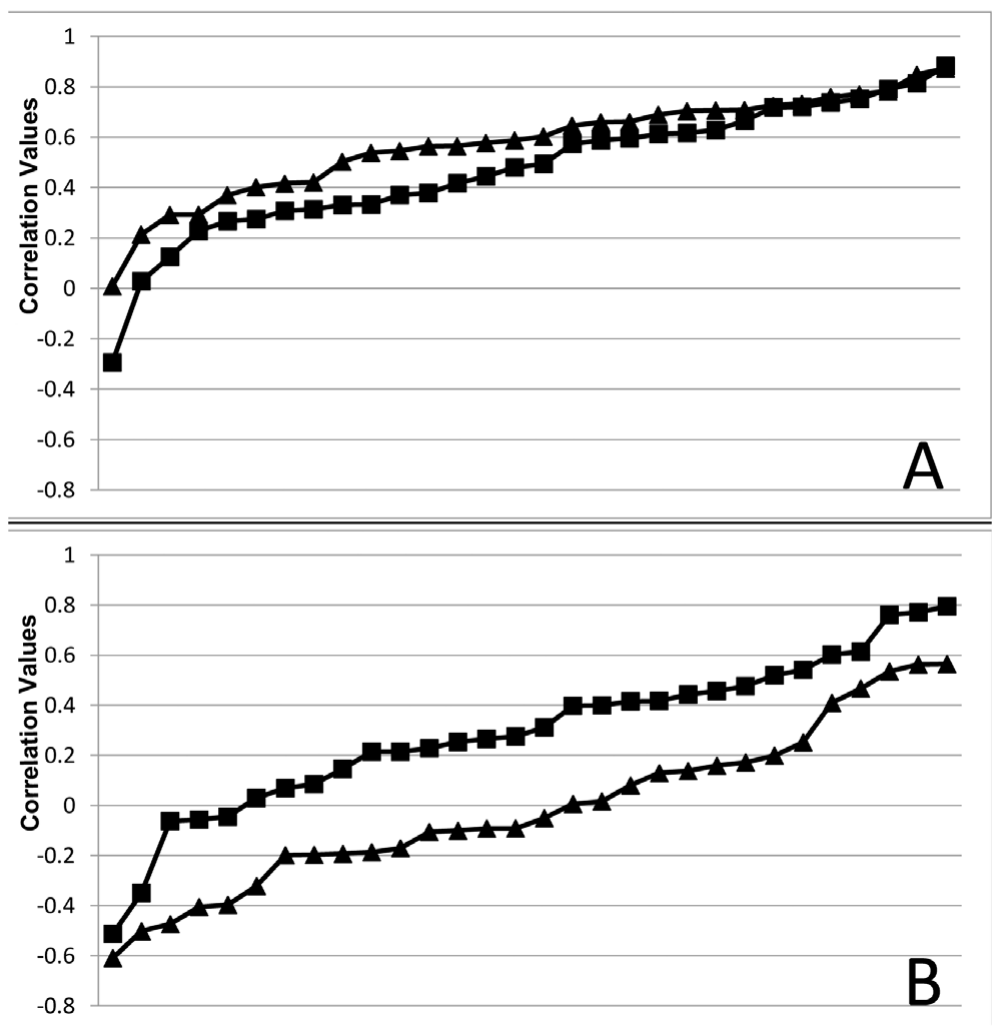
Correlation distributions in LV1 and LV2. Correlation values for all 10-sec blocks, sorted and plotted from lowest (most negative) to highest (most positive), to show the distributions in LV1 and LV2. A) LV1 – Rest1 correlations (squares) are not significantly different from Rest2 correlations (triangles). B) LV2 – Rest1 correlations (squares) are more positive than Rest2 correlations (triangles).

To assess whether the distribution of correlations differed between Rest1 and Rest2, we used a non-parametric test to compare the correlations seen in Figures 1 and 2. As seen in Figure 3, these distributions were not found to be significantly different for LV1 (z = −1.4, p = 0.16, Wilcoxon test), but did differ for LV2 (z = −3.48, p = 0.0005). That is, the pattern of PCC connectivity seen in LV1 was equally positive for both rest runs, whereas in LV2 the PCC was more connected with other DN regions (more positive correlations) in Rest1 and more connected with the alternate regions during Rest2 (more negative correlations).

### Stable vs. variable DN spatial patterns

The next step was to elaborate and differentiate between stable and variable DN regions; we use “stable” to refer to areas that showed positive network connectivity with other DN regions on both LV1 and LV2, and “variable” to describe those regions with different connectivity on LV1 and LV2. To do this, we created two conjunction maps.

**Figure 4:**
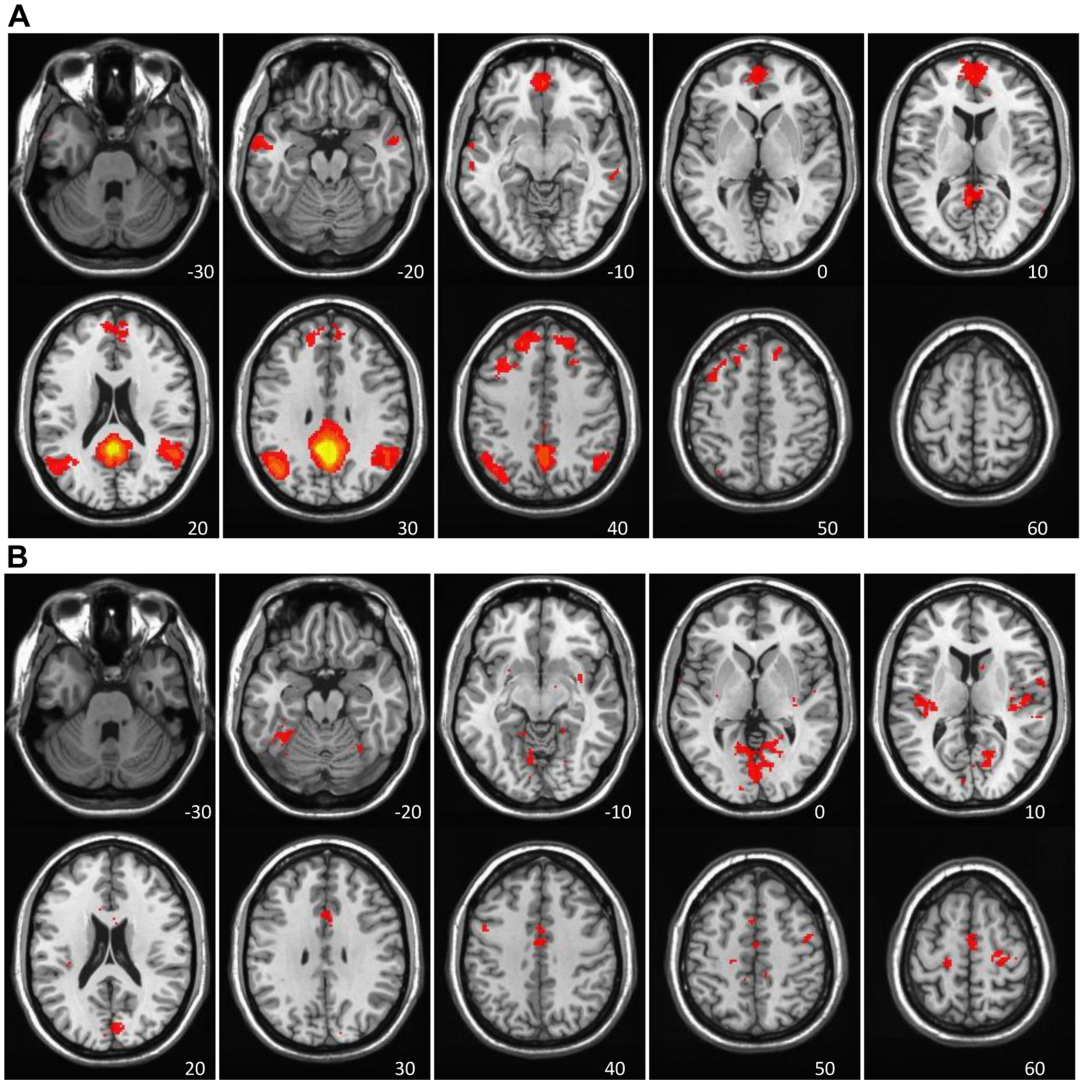
Conjunction analyses of LV1 and LV2. Conjunction analyses of LV1 and LV2, highlighting stable and variable DN regions. A) Stable DN regions – voxels showing positive brain scores, and positive FC, on both LVs (red regions in both Figure 1 and 2). B) Variable DN regions – voxels with positive brain scores (positive DN connectivity) on LV1 (red regions in Figure 1) and negative brain scores (negative DN connectivity) on LV2 (blue regions in Figure 2). The colors represent the conjunction “BSR” values, which are a product of the BSR values of the original maps, thereby highlighting common “hotspots”.

The first conjunction map identified DN regions common to both LVs, i.e. - voxels that had positive BSRs (red regions seen in Figures 1 and 2) in both (Figure 4a). This map, isolating the regions that showed stable FC with the PCC in both LVs, included most of the areas currently thought to comprise the DN, such as medial frontal gyrus, angular gyrus and middle temporal gyrus (see Table 3a for the entire list). Notably absent from this common map were the medial temporal regions and cerebellum.

**Table 3:** Brain areas identified with conjunction analyses.

Region	Hem	X(mm)	Y(mm)	Z(mm)
**3a - regions showing stable FC with the PCC, in both LVs**				
Angular gyrus	left	−48	−62	35
Inferior parietal lobule	left	−50	−57	46
Medial frontal gyrus	midline	1	45	−12
Medial frontal gyrus	right	8	56	22
Middle cingulate cortex	midline	4	−26	36
Middle frontal gyrus	left	−37	30	43
Middle temporal gyrus	left	−65	−3	−23
Posterior cingulate cortex	midline	1	−46	18
Posterior cingulate cortex	right	14	−47	29
Precuneus	midline	−1	−49	31
Precuneus	right	6	−63	44
Superior frontal gyrus	left	−16	49	34
Superior frontal gyrus	right	23	49	38
Superior temporal gyrus	right	55	−58	29
Thalamus	midline	1	−16	17
**3b - regions showing variable FC with the PCC, in LV2**				
Anterior cingulate cortex	right	8	14	24
Cuneus	left	−7	−62	3
Cuneus	right	6	−92	8
Cuneus	right	16	−74	15
Fusiform gyrus	left	−40	−47	−24
Insula	left	−31	−24	9
Lingual gyrus	left	−12	−49	−13
Lingual gyrus	midline	1	−86	1
Lingual gyrus	right	12	−56	0
Middle cingulate cortex	right	10	21	36
Precentral gyrus	right	23	−21	62
Precentral gyrus	right	67	0	6
SMA - BA6	midline	4	−5	54
Superior frontal gyrus	right	8	14	57
Superior frontal gyrus	right	25	−15	66
Superior temporal gyrus	left	−56	−24	5
Superior temporal gyrus	right	57	−21	2

MNI coordinates. BSR>3.3 is equivalent to p<0.001. Hem = hemisphere; SMA = supplementary motor area; PCC = posterior cingulate cortex, the seed used in the FC analysis. See also Figure 4.

The second map (Figure 4b) identified voxels that were part of the FC pattern seen throughout Rest1 and Rest2 in LV1 (red regions in Figure 1) but more variably correlated with other DN areas in LV2 (blue regions in Figure 2). This map included areas such as anterior cingulate, lingual gyrus, superior temporal gyrus, and precentral gyrus (see Table 3b for the entire list). These two maps shown in Figure 4 therefore identify two subsets of regions that are functionally connected to the PCC, one of which shows positive connectivity regardless of whether FC is assessed before or after a series of cognitive tasks, and a second that shows more variable connectivity after participants carry out cognitive tasks.

### Contrasts Exploring FC differences in rest and task runs

**Figure 5:**
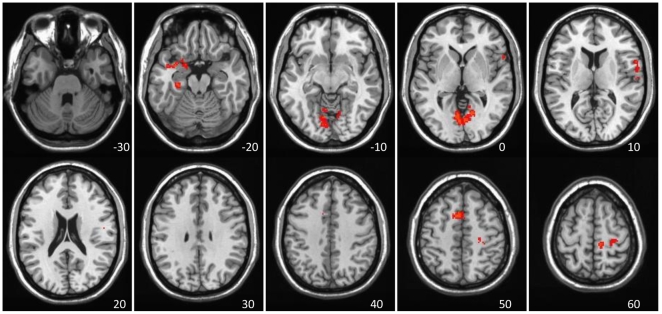
Direct comparison of Rest1 and Rest2. Areas showing greater FC with PCC in Rest2 than Rest1, as found in the contrast analysis, and shown in red (BSRs>3.3). No negative BSRs met the threshold.

The direct comparison of Rest1 and Rest2, across all time points, is shown in Figure 5 (p<0.002). This contrast identified only areas with stronger connectivity during Rest2 (there were no above-threshold regions with stronger connectivity in Rest1). There was stronger connectivity during Rest2 in supplementary motor area (SMA), precentral and postcentral gyri, lingual gyrus, and amygdala (see Table 4a for a full list). Not surprisingly, these areas overlap with those seen in LV2 of the previous analysis, specifically with areas that showed more variable FC with the PCC and other DN regions (see Figure 4b). This suggests that the increased connectivity with these regions from Rest1 to Rest2 is due to an influence of the intervening tasks. However, it is important to separate differences in FC that might reflect being in the scanner for some length of time from those that are due to the influence of carrying out cognitive tasks.

**Table 4:** Brain areas identified with contrast-driven analyses.

Region	Hem	X(mm)	Y(mm)	Z(mm)	BSR
**4a - regions showing stronger PCC FC in Rest2, relative to Rest1**					
Amygdala	left	−22	−4	−26	−4.75
Lingual gyrus	left	−8	−72	−8	−5.88
Paracentral lobule	right	10	−20	68	−5.90
Parahippocampal gyrus	left	−26	−24	−24	−4.82
Postcentral fyrus	right	52	−10	16	−5.23
Precentral gyrus	right	28	−18	46	−5.26
SMA - BA6	midline	−4	12	50	−4.99
Superior temporal gyrus	left	−34	2	−20	−4.76
**4b - regions showing stronger PCC FC in Rest2 & Task8, relative to Rest1 & Task1**					
Fusiform gyrus	left	−32	−42	−12	−5.96
Lingual gyrus	left	−4	−66	4	−4.80
Precuneus	right	14	−58	46	−5.04
Precuneus	left	−10	−54	44	−4.45
SMA - BA6	left	−4	12	50	−5.47
**4c - regions showing stronger PCC FC in Rest2, relative to Rest1, as well as weaker FC in Task8 relative to Task1**					
Medial frontal gyrus	Right	8	−24	66	−5.16
Paracentral lobule	Left	−18	−20	56	−6.15
Precentral gyrus	Right	60	8	10	−4.86
Supramarginal gyrus	Right	52	−50	36	−5.44

MNI coordinates. BSR>3.3 is equivalent to p<0.001. Hem = hemisphere; SMA = supplementary motor area; PCC = posterior cingulate cortex, the seed used in the FC analysis. See also Figure 5+6+7.

**Figure 6:**
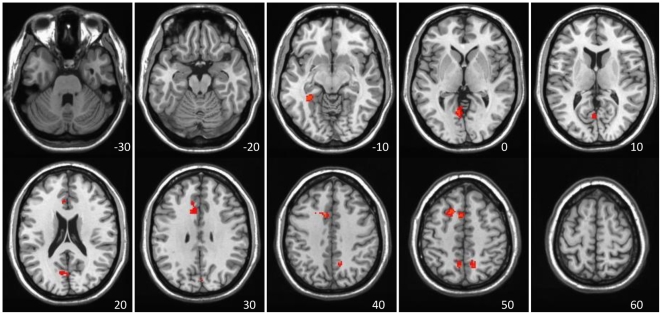
Direct comparison of Rest1 & Task1 vs. Task8 & Rest2. Areas showing greater FC with PCC in Rest2 and Task8, compared to Rest1 and Task1, and shown in red (BSRs>3.3). No negative BSRs met the threshold.

To address this issue, we tested for common differences between FC in the two rest conditions and the first and last task runs, as well as differences unique to the two rests. The first contrast identified those areas with similar changes between the first and second rest runs and the first and last task runs, i.e., those changes likely due to time in the scanner. These areas are shown in Figure 6 and are limited to five clusters in bilateral precuneus, left fusiform gyrus, SMA, and lingual gyrus (p = 0.001). In all of these areas there was stronger FC during Rest2 and Task8, relative to Rest1 and Task1 (Table 4b; there were no regions with stronger FC during Rest1 and Task1). Therefore, changes in FC here could be due to the effect of time in the scanner.

**Figure 7:**
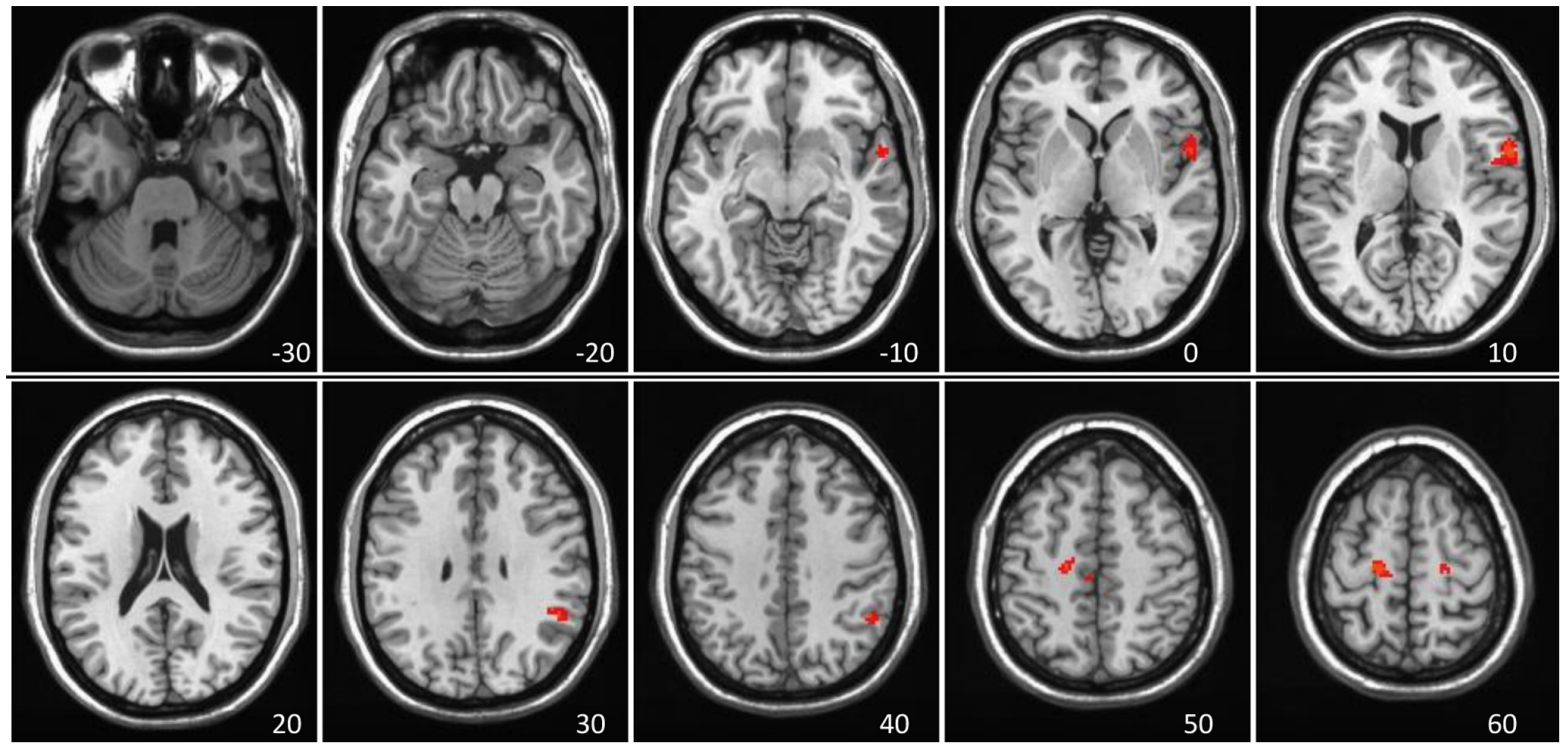
A time×run interaction analysis of Rest1/Task1/Task8/Rest2. Areas showing stronger FC with PCC in Rest2 relative to Rest1, and weaker FC in Task8 relative to Task1, as found in the interaction contrast analysis, and shown in red (BSRs>3.3). No negative BSRs met the threshold.

Finally, a contrast to identify areas with a time×run type interaction was carried out. This is a stringent test of those effects limited to the difference between Rest1 and Rest2 because it requires the areas that increase in strength between the resting runs to weaken between the two task runs. This contrast (p = 0.004) showed a set of regions with stronger PCC connectivity in Rest2, relative to Rest1, but weaker connectivity in Task8 relative to Task1 (Figure 7). These were the left paracentral lobule, right medial frontal gyrus, precentral gyrus, and supramarginal gyrus (Table 4c).

Most of the regions identified by these direct contrasts were also identified in the second LV of the data-driven analysis. This suggests that the observed overall FC change in these regions, with the exclusion of regions identified as showing stronger FC in both rest and task runs (Figure 6), is likely due to an influence of cognitive processing on resting activity in the second resting-state run, and not just time spent in the scanner.

## Discussion

The study of the DN, and the brain states that it supports, is expanding in recent years. However, two possibly fundamental aspects of the behavior of this network have been relatively understudied. One is the temporal nature of the functional interactions among nodes of the network, and the second is whether DN functional connectivity can be influenced by a preceding brain state. In our study, we aimed to address the possibility of variability in both spatial and temporal domains. We compared the resting-state FC of the DN using two runs that were separated by a 45 minute interval containing task execution. We found that the DN is not temporally static, but can vary dynamically over time. The results revealed two primary modes of FC as assessed using the PCC as a seed – a robust correlation among DN regions, and a switching between connectivity among certain DN regions and connectivity among other areas, including some task-positive regions. The first FC pattern represents a stable feature of the DN, suggesting that the DN indeed maintains some temporally stable functional connections. However, the second FC pattern may represent a dynamic behavior of certain DN regions that occurs during rest periods that follow tasks, suggesting an interaction between task-positive regions and DN regions that carries over into resting-state periods.

Therefore, the DN seems to exhibit two *simultaneous* FC dynamics at rest, one robust (accounting for 36% of data covariance) and one secondary (6% covariance). The first is stable in time and insensitive to previous brain states. The set of regions showing this stable pattern of FC involved all regions currently thought to be part of the DN [5], [7], [8], as well as some additional areas not currently included in the DN, but that we have previously shown are strongly functionally connected to the PCC [29]. We show here that this widely distributed set of positive correlations is the most prominent FC pattern for the PCC, and does not include any anti-correlated regions. The second dynamic is more variable, does include some regions that are anti-correlated with a subset of the DN seen in the first FC pattern, and is seen more strongly when the resting-state follows a period of task execution, suggesting it may be an after-effect of the cognitive activity engaged during the tasks. The set of DN regions that correlated with the PCC on this pattern are similar to the set typically reported for the DN, minus a few areas like the medial temporal lobes. The set of areas comprising the alternate group that correlated with the PCC, and negatively correlated with the DN regions, included some general TPN areas, such as the parietal areas and SMA, and the anterior insula/inferior frontal regions. These two FC patterns that switch back and forth during Rest2 may indicate that participants fluctuate between internal thoughts (mediated by the DN [29], [44]) and monitoring of the external environment (TPN areas, occipital cortex [7], [8]) and somatic state (posterior insula [45]). We suggest two conclusions from this result. Firstly, the DN that most studies have described may be the more variable subset that we see in LV2, suggesting that the strongest FC pattern of the PCC is more widely distributed. Secondly, we were only able to see multiple co-existing patterns by using a technique that allows for this. Future studies should explore this idea further for the DN as well as other resting state networks.

A few recent studies suggest that the DN is spatially stable at rest, across time spans of minutes, hours, or even months [22], [46], [47], but also sensitive to seemingly minor differences between the conditions in which it is studied, such as whether the resting run is carried out with eyes open vs. closed [48]. Our work similarly suggests that the DN is reliable from one measurement to another, but nevertheless can reflect some perturbations due to outside influences, or perhaps internal ones as well. This interesting characteristic is clearly demonstrated in our study, which specifically investigated differences between pre- and post-task resting states. Our results indicate that reliability of the DN and its sensitivity to intervening influences may be seen in different aspects of its functional connectivity. That is, the primary FC dynamic reflects DN reliability and stability, and is not sensitive to the previous task state, whereas the secondary dynamic reflects sensitivity to task effects.

There was consistency between the FC patterns identified in LV2 of the data-driven analysis and the subsequent contrast analyses in terms of the regions showing different FC between Rest1 and Rest2. For example, both LV2 and the direct contrast of Rest1 and Rest2 showed more FC for the left medial temporal region, medial frontal/SMA, and lingual gyrus during the post-task resting run. The contrasts comparing FC during rest and task runs suggested that strengthened FC in the lingual gyrus and medial frontal areas might be a consequence of time spent in the scanner, whereas stronger FC in SMA, anterior temporal regions and right supramarginal gyrus is more likely to be an influence of intervening task on resting FC. Some of these regions that exhibited greater FC with the PCC in Rest2, or more variable FC in Rest2, also exhibited increased activity (compared to baseline) during internally-oriented tasks (SMA, inferior frontal gyri) or during externally-oriented tasks (right supramarginal gyrus) in our previous study [29]. Although this suggests some specific influences of task demands on resting FC of the DN, future studies limiting the type of intervening cognitive processes will be necessary to determine if the effect that we observed on resting FC is a product of our specific tasks, or of general task execution per se. There also is a possibility that Rest2 was influenced by Rest1 itself, as others have found [48], but we cannot address it here given the intervening task runs. Regardless, it is likely that any possible order effect of the resting runs per se would be outweighed by the task-related effect we observed.

It is unlikely that the carry-over we observed is a product of the sluggish temporal nature of the BOLD signal, especially since we observed it as a secondary dynamic to the more robust, stable pattern of FC. In addition, this carry-over is unlikely to constitute simple residual processing from the previous task-induced brain state, since it was present off and on during the entire second resting scan. If it had been residual task-related processing, it would have probably exhibited a “recovery” behavior, similar to that shown in Barnes et al's study [26]. More likely, this dynamic may represent an interaction between DN regions (involved in internally-oriented cognition) and TPN regions (involved in externally-oriented cognition), as well as regions more specific to the preceding active brain state (depending on the specific task executed). In some cases, this interaction, in turn, might reflect post-task consolidation or learning [44], [49].

Finally, our study highlights some practical issues which should be taken into consideration in future work involving resting-state FC. First, our observation of different DN FC patterns between pre- and post-task resting-states shows that not all “rests” are the same. From a practical standpoint, researchers should take note that FC calculated from a resting run that follows some cognitive activity may be influenced by this previous brain state. A particularly striking example is our observation that the PCC is more strongly functionally correlated with some TPN regions after a series of tasks has been performed. Moreover, this influence might not necessarily just be carried over to a post-task resting-state, but also to any post-task state, be it rest or a new task. Therefore, a previous task might impact brain activity during the performance of a current task, something which is rarely if ever assessed.

Second, the fact that we observed two simultaneous resting-state dynamics highlights the possibility that brain networks/areas may be involved in multiple processing modes at any given point in time. This notion is consistent with the idea of neural context [50] and is an indication that network FC is both fluid and complex. This observation of multiple dynamic aspects of FC of a single region, the PCC in this case, may also characterize brain function more generally and reflect a fundamental aspect of functional organization in the brain. It is important to note that this result was made possible by the use of a multivariate data-driven approach, which was both less constrained than model-driven ones, and well-suited to capture the complex nature of brain function.
